# KCC2 downregulation facilitates epileptic seizures

**DOI:** 10.1038/s41598-017-00196-7

**Published:** 2017-03-13

**Authors:** Lulan Chen, Li Wan, Zheng Wu, Wanting Ren, Yian Huang, Binbin Qian, Yun Wang

**Affiliations:** Institutes of Brain Science, State Key Laboratory for Medical Neurobiology, Collaborative Innovation Center for Brain Science, Fudan University, Shanghai, 200032 China

## Abstract

GABA_A_ receptor-mediated inhibition depends on the maintenance of low level intracellular [Cl^−^] concentration, which in adult depends on neuron specific K^+^-Cl^−^ cotransporter-2 (KCC2). Previous studies have shown that KCC2 was downregulated in both epileptic patients and various epileptic animal models. However, the temporal relationship between KCC2 downregulation and seizure induction is unclear yet. In this study, we explored the temporal relationship and the influence of KCC2 downregulation on seizure induction. Significant downregulation of plasma membrane KCC2 was directly associated with severe (Racine Score III and above) behavioral seizures *in vivo*, and occurred before epileptiform bursting activities *in vitro* induced by convulsant. Overexpression of KCC2 using KCC2 plasmid effectively enhanced resistance to convulsant-induced epileptiform bursting activities *in vitro*. Furthermore, suppression of membrane KCC2 expression, using shRNA_KCC2_ plasmid *in vitro* and shRNA_KCC2_ containing lentivirus *in vivo*, induced spontaneous epileptiform bursting activities *in vitro* and Racine III seizure behaviors accompanied by epileptic EEG *in vivo*. Our findings novelly demonstrated that altered expression of KCC2 is not the consequence of seizure occurrence but likely is the contributing factor.

## Introduction

GABA_A_ receptor is a Cl^−^ permeable ionotropic receptor, which can mediate either excitatory or inhibitory synaptic responses depending on the intracellular Cl^−^ concentration ([Cl^−^]_i_)^1, 2^. The [Cl^−^]_i_ is largely determined by the *SLC12A* cation-chloride cotransporters, including the sodium-potassium-chloride cotransporter 1 (NKCC1) and the neuron-specific potassium-chloride cotransporter 2 (KCC2). In immature neurons, robust expression of NKCC1 is regarded as a major source of Cl^−^ inner transporter to maintain a high [Cl^−^]_i_. Due to developmental upregulation of KCC2 expression but downregulation of NKCC1 in mature neurons, KCC2 is the primary determinant in maintaining [Cl^−^]_i_ below electrochemical equilibrium by extruding Cl^−^^3–5^. Robust KCC2 expression is essential to promote the switch from excitatory to fast inhibitory action of GABA during development^3^. Decrease of KCC2 expression level and subsequent diminish of GABA inhibitory effect has been found in several neurological disorders, including epilepsy^6–13^. Epilepsy is known as the recurrence of seizures, and seizure is defined as abnormal excessive or synchronous neuronal network activities^14^. The decrease of GABA inhibition leading to the imbalance of excitation and inhibition will generate the seizure^15, 16^. Subicular pyramidal neurons from patients with refractory temporal lobe epilepsy exhibit positive shift of GABA responses^17^, which are attributable to reduction of KCC2 expression^17, 18^. And KCC2 downregulation is also detected in the hippocampus of temporal lobe epilepsy animal models, giving rise to inhibitory efficacy reduction and neuron excitability enhancement^12, 13^.

Given the crucial role of KCC2 in maintaining GABA_A_R inhibitory function, understanding the casual link between KCC2 downregulation and seizure is particularly important. In previous studies KCC2 downregulation was observed after epilepsy. Several researches have found that KCC2 downregulation is triggered by abnormal excitatory activities of neuronal network^19^. Elevated activity induced by glutamate application caused downregulation of KCC2 and this downregulation is dependent on Ca^2+^-permeable NMDA receptor activity^20^. However, it is still unclear whether KCC2 was downregulated before seizure or as a consequence of seizure.

To further figure out the temporal relationship between the downregulation of KCC2 and the seizure, we combined electrophysiology and molecular biology approaches to demonstrate that KCC2 downregulation preceded convulsant induced epileptiform bursting activities both i*n vitro* and *in vivo*. Intervening endogenous KCC2 expression level in cultured hippocampal neurons directly resulted in increase of spontaneous epileptiform bursting activity, while overexpression of KCC2 increased the resistance to convulsant induced epileptiform bursting activity. Furthermore, seizure-like behaviors and epileptiform EEG have been observed in individual rat, in which KCC2 was knocked down in dentate gyrus. Our studies first time indicated that the activity dependent reduction of KCC2 expression and function contributed to the development of epileptic seizure.

## Results

### KCC2 reduction is associated with animal epileptic seizures *in vivo*

**Figure 1:**
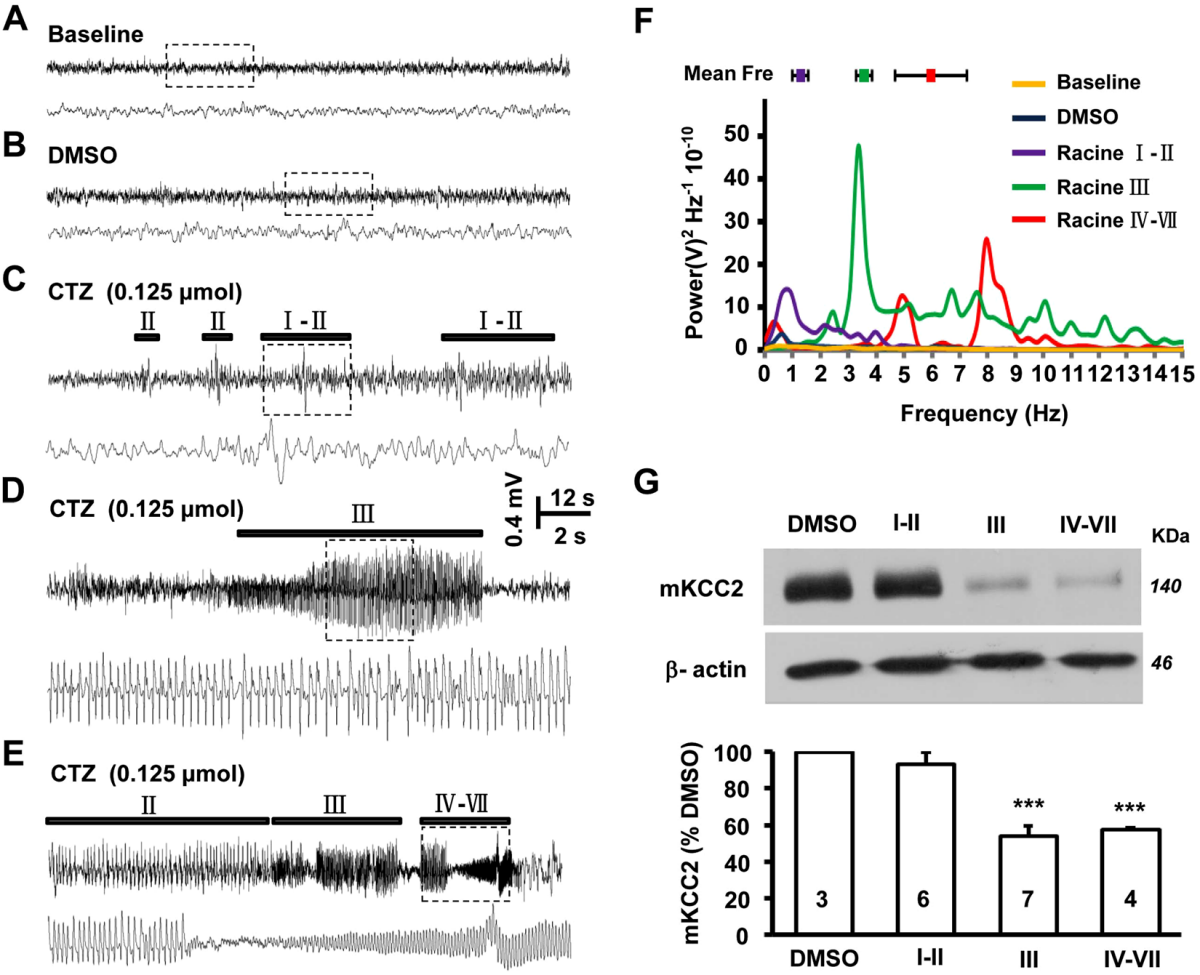
Epileptiform HFHA EEG correlated to seizure behavior dependent change of membrane KCC2 in CTZ-induced seizure animals. (**A–E**) Representative traces of EEG recordings from rats with different Racine score seizure behaviors induced by i.c.v CTZ injection. DMSO served as vehicle control. No abnormal EEG activity was observed before injection (**A**) or after DMSO injection (**B**). CTZ-induced either single low frequency spike discharge (**C**) along with MRS I-II seizure behaviors (Supplementary video 1a), or high frequency and high amplitude (HFHA) bursting activities (**D**) associated with Racine III (Supplementary video 1b), or HFHA bursting activities (**E**) associated with Racine IV-VII seizure behaviors (Supplementary video 1c). (**F**) Sample power spectrum analysis of the representative EEG recordings from (**A–E**), and the mean peak power frequency analysis associated with MRS I-II, III or IV-VII behaviors. (**G**) Representative cropped western blot results showing the quantity of membrane KCC2 in different seizure level animals. The gels were run under the same experimental conditions and were cropped around 140 and 46 KDa. The full-length gels were presented in Supplementary Fig. 2. Bar histogram showing quantification of KCC2 expression in different seizure level animal groups. CTZ sub-groups were all normalized to DMSO group. KCC2 was significantly downregulated in Racine III (***P < 0.001) and IV-VII score animals (***P < 0.001).

Cyclothiazide (CTZ) is a potent convulsant by which we established both *in vivo* and *in vitro* epilepsy model in our previous studies^21, 22^. Compared with KA or other commonly used convulsants, lower toxicity and longer latency is revealed in CTZ-induced epileptiform activities^23, 24^. Previous studies suggested that membrane KCC2 was downregulated in epileptic seizures^25, 26^, but the relationship between seizure behavior and membrane KCC2 expression is still unclear. In this study, we monitored CTZ-induced seizure activities in freely moving rats using behavior observation simultaneously recorded EEG activities and then, 24 hours later, examined the membrane KCC2 level in animal groups with different seizure stages by western blot. Consistent with our previous data^21^, DMSO group (N = 3) did not show any seizure behaviors or epileptic EEG activities (Fig. 1A,B). However, injection of sub-maximal dose of CTZ (0.125 μmol in 5 μl, i.c.v.) induced seizure behaviors with various maximum seizure levels and EEG discharges in tested animals. In total of 17 CTZ treated animals, 35.3% (N = 6) only displayed maximal MRS I-II seizure behaviors (Supplementary video 1a) which were only accompanied with the single low-frequency interictal-like spike discharges (Fig. 1C), in addition, 41.2% (N = 7) and 23.5% (N = 4) animals showed maximal MRS III (Supplementary video 1b) and IV-VII seizure behaviors (Supplementary video 1c), respectively, which were directly associated with high-frequency-and-high-amplitude (HFHA) ictal-like bursting EEG activities (Fig. 1D,E). Further power spectrum analysis revealed a seizure behavior level related increase of the peak power frequency of those HFHA epileptiform activities (Fig. 1F). The mean peak power spectrum frequency of seizure events was 1.3 ± 0.3 Hz (n = 103, N = 5), 3.6 ± 0.3 Hz (n = 60, N = 7) and 6.0 ± 1.3 Hz (n = 21, N = 3) in MRS I-II, III and IV-VII animal groups, respectively (Fig. 1F). Importantly, further WB results showed that the membrane KCC2 expression level in hippocampus was only significantly decreased in animals with MRS III (57.3 ± 5.2% of the DMSO group, N = 7; P < 0.001) and MRS IV-VII (57.6 ± 1.1%, N = 4; P < 0.001), but not in MRS I-II animal group (93.4 ± 6.5%, N = 6; P = 0.38) (Fig. 1G).

These *in vivo* experiment results indicated that the epileptiform bursting activities were directly associated with the higher level seizure behaviors, and positively correlated to a significant downregulation of plasma membrane KCC2 level.

### KCC2 reduction occurred before the epileptiform bursting activities

**Figure 2:**
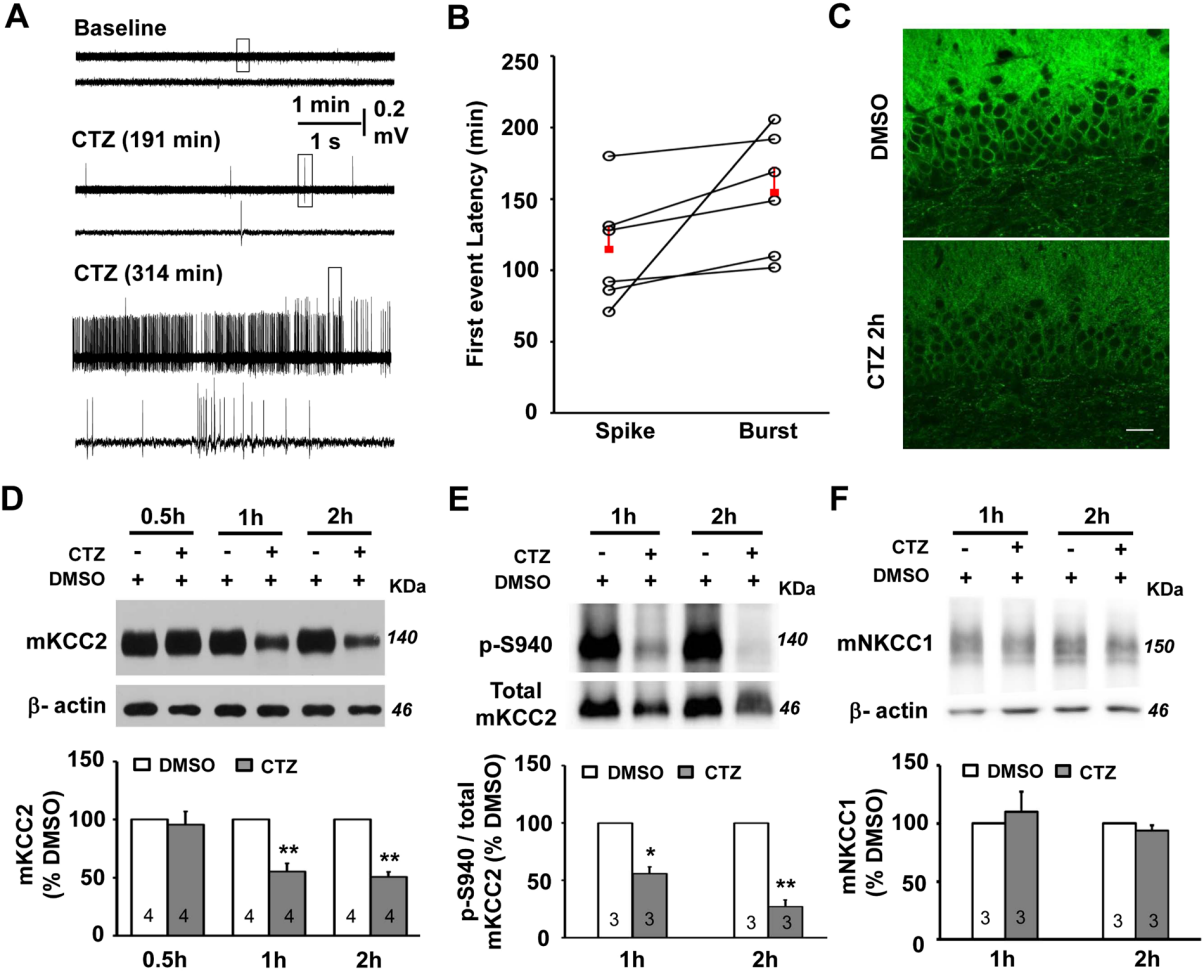
Change of KCC2 expression involved in the progress of epileptiform activity in hippocampal slices. (**A**) Extracellular field potential recording of epileptiform activities in DG granule cell layer induced by 50 μM CTZ. Sample traces showing baseline (upper), interictal-like spike discharge (middle) or HFHA bursting activity (bottom) at different time points after CTZ treatment. (**B**) Dot plot showing the individual and the group data of the latency of interictal-like spike or ictal-like bursting activities after CTZ treatment. (**C**) Immunostaining images showing the downregulation of KCC2 in DG after CTZ treatment for 2 hr. Scale bar, 20 μm. (**D,E**) Western blot results showing the change of membrane KCC2 expression at 0.5, 1 and 2 hr (**D**), or membrane pS940 KCC2 at 1 and 2 hr (**E**) after CTZ or DMSO treatment, respectively. The gels were run under the same experimental conditions and were cropped around 140 and 46 KDa. The full-length gels were presented in Supplementary Figs 3 and 5. Bar histogram showing the quantification of KCC2 expression or value of membrane pS940/total membrane KCC2 normalized to control. Membrane KCC2 or pS940/membrane KCC2 level was significantly downregulated at 1 hr (*P < 0.05, **P < 0.01) or 2 hr time point (**P < 0.01). (**F**) Sample western blot showing membrane NKCC1 expression was low and no significant change at 1 and 2 hr after CTZ treatment. The gels were run under the same experimental conditions and were cropped around 140 and 46 KDa. The full-length gels were presented in Supplementary Fig. 6. Bar histogram showing the quantification of NKCC1 expression normalized to control.

To understand the role of KCC2 in the seizure induction, we further studied the temporal relationship between the epileptiform bursting activities and the downregulation of KCC2 by using *in vitro* hippocampal slice preparation. Bath application of either CTZ (50 μM) induced a progressive increase of neuronal network activities in dentate gyrus layer, which gradually developed to recurrent interictal-like spike activities and later transformed to highly synchronized ictal-like HFHA bursting discharges (Fig. 2A). Among 6 slices showed epileptiform bursting firing induced by CTZ, the average latency of the first interictal-like spike and ictal-like bursting activity was 114.7 ± 16.3 min (range: 71 to 180 min) and 154.7 ± 17.4 min (range: 102 to 206 min), respectively (Fig. 2B). In association with this activity increase, we found the immunoreactivity of KCC2 labeling has already been significantly decreased in the dendritic area of the granule cells in DG (63 ± 7%, normalized to DMSO, n = 3; one sample T test, P < 0.05) at 120 min (2 h) after CTZ treatment (Fig. 2C). In order to further reveal the temporal relationship between the epileptiform bursting activities and the downregulation of the membrane KCC2 expression, WB experiments were performed to detect the membrane KCC2 expression change at the time point of 0.5, 1 and 2 hr after CTZ treatment. The results in CTZ model showed that there was a pronounced fall of the plasma membrane KCC2 protein level started between 0.5 to 1 hr time point (55.1 ± 7.2%, n = 4; P < 0.01), through out to the 2 hr time point (50.8 ± 4.2%, n = 4; P < 0.01) (Fig. 2D). In addition, in acute 0-Mg^2+^ model, a well acknowledged *in vitro* epilepsy model, similar epileptiform activities were evoked (Supplementary Fig. 1A) with the average latency of the first interictal-like spike and the ictal-like HFHA bursting discharge was 44.8 ± 12.2 min (range: 21 to 76 min, n = 5) and 145.7 ± 23.0 min (range: 86 to 228 min, n = 5), respectively (Supplementary Fig. 1B). However, the significant decrease of the plasma membrane KCC2 level was detected as early as at 1 hr time point (68.1 ± 6.2%, n = 4; P < 0.05) and through out to 2 hr time point (56.5 ± 2.9%, n = 3; P < 0.01) of time-matched normal ACSF control (Supplementary Fig. 1C). Compared to the time point between the bursting activities and the membrane KCC2 downregulation in the same preparation from either CTZ or 0-Mg^2+^ model, it was interesting to find that the membrane KCC2 downregulation occurred along with the increased hippocampal activity but much earlier than the happening time point of the epileptiform bursting activities.

Phosphorylation of KCC2 residue S940 plays an important role in maintaining surface KCC2 stability and activity^20^. Our WB results further revealed that the ratio of the membrane pS940 KCC2/total membrane KCC2 protein was significantly declined as early as at 1 hr (55.9 ± 5.8%, n = 3; P < 0.05) and with further reduction (27.2 ± 5.8%, n = 3; P < 0.01) at 2 hr time point, indicating a significant dephosphorylation of S940 occurred as early as at 1 hr after CTZ treatment in hippocampal slices (Fig. 2E). Besides KCC2, the membrane expression of NKCC1 in hippocampus was also examined in the *in vitro* CTZ model. Our results showed that the expression level of NKCC1 was rather very low, almost undetectable in both normal control and CTZ treatment condition. In this experiment condition, the quantification of the expression level of NKCC1 after CTZ treatment at either 1 h or 2 hr time points showed no significant change (1 h: 109.9 ± 17.4%, n = 3; 2 h: 93.8 ± 4.7%, n = 3) compared to DMSO control treatment (Fig. 2F).

These results showed that the downregulation of the membrane KCC2, as well as the reduction of the ratio of pS940 membrane KCC2, the stable phase of the membrane KCC2, during seizure induction preceded the epileptiform bursting activities, suggesting that it might be the cause for epileptiform bursting activity generation.

### Dysfunction of KCC2 occurred before the epileptiform bursting activities

Above results indicated that the significant loss of membrane KCC2 and its S940 residue phosphorylation had occurred between 0.5 to 1 hr and lasted to at least 2 hr after CTZ treatment. To test whether CTZ-induced membrane KCC2 loss also affected its function, the direct measurement of Cl^−^ extrusion capacity and *E*_GABA_ was performed in CA1 pyramidal neurons in hippocampal slices.

**Figure 3:**
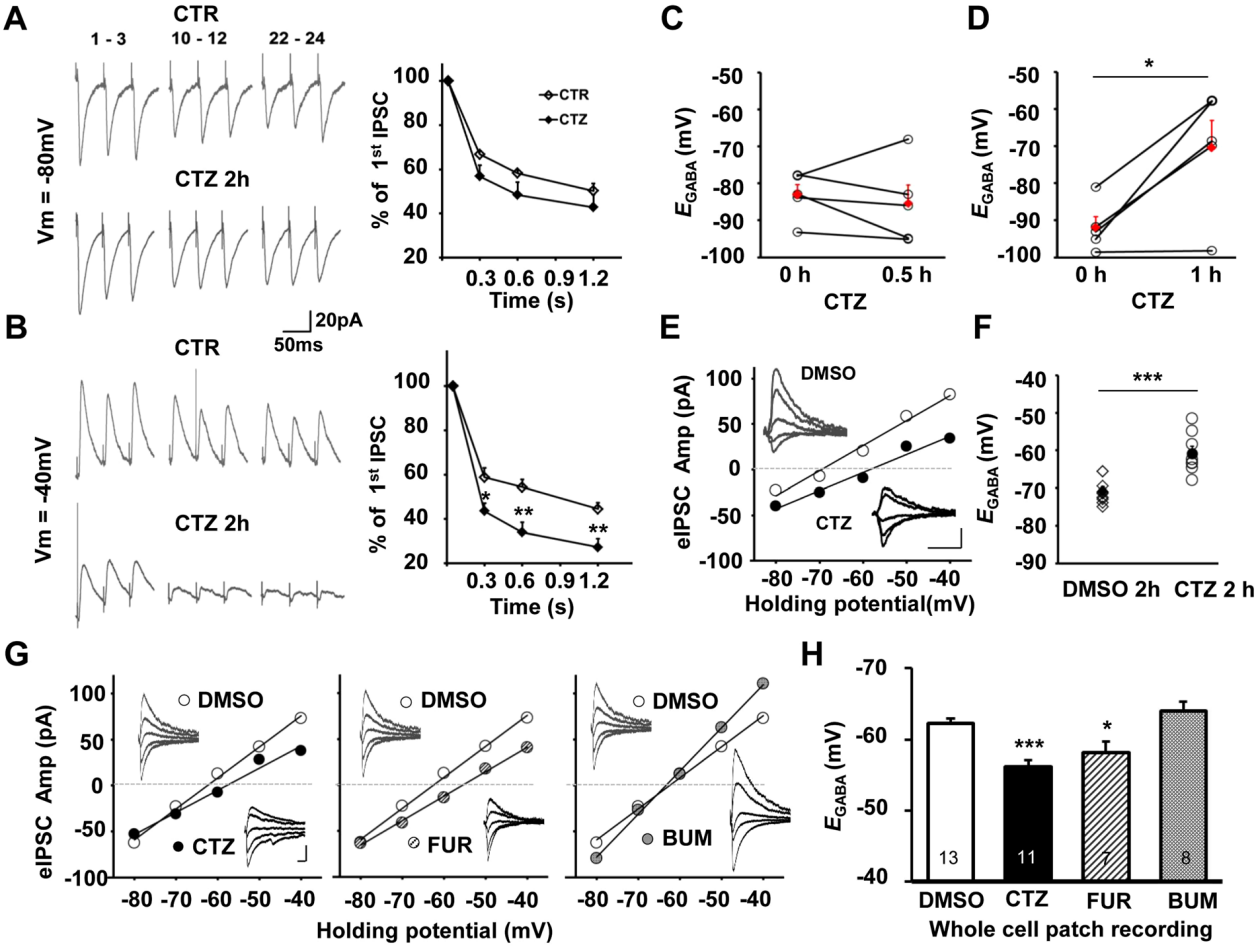
The function of KCC2 was impaired before the epileptiform bursting activities *in vitro*. (**A,B**) Representative traces showing evoked GABAergic currents were recorded at either −80 mV (A left) or −40 mV (B left) during HFS (1.2 s, 20 Hz) under control and CTZ conditions. Line chart showing the synaptic depression with little change under −80 mV condition (A right), but significantly impaired under −40 mV condition (B right). (**C,D**) *E*_GABA_, determined from same neuron in hippocampal slices at either 0 and 0.5 hr (**C**) or 0 and 1 hr (**D**) with 50 μM CTZ treatment, was plotted and the mean value was shown in red. No obvious change of *E*_GABA_ after 0.5 hr (P = 0.70), but a significant depolarizing shift of *E*_GABA_ after 1 hr CTZ treatment (*P < 0.05) was observed. (**E,F**) Example recordings of *E*_GABA_ by gramicidin perforated whole cell patch clamp after 2 hr incubation of either DMSO or 50 μM CTZ. Dot plot indicated that *E*_GABA_ was significantly positive shifted after 2 hr treatment of CTZ (***P < 0.001). (**G**) I-V plots showing the experimental determination of the *E*_GABA_ after either CTZ, furosemide or bumentanide treatment under whole cell recording condition. (**H**) Bar histogram showing that a significantly depolarization shifts of *E*_GABA_ with either CTZ (***P < 0.001) or furosemide (*P < 0.05), but not with bumentanide treatment.

First we tested whether CTZ-induced membrane KCC2 loss directly affected its sustained Cl^−^ regulatory capacity. Repetitive stimulation of Schaffer collateral evoked frequency dependent synaptic depression on recorded GABAergic IPSCs in CA1 pyramidal neurons under gramicidin perforation patch clamp, similar as previous reported^27^. At the holding potential of −80 mV, which is favored for Cl^−^ efflux through GABA_A_ receptors, frequency dependent IPSC depression induced by a train of stimulation (24 pulses at 20 Hz) had no significant difference between DMSO control and 2 hr CTZ treatment groups (n = 8; P > 0.05; Fig. 3A). This result suggested that no matter whether membrane KCC2 was downregulated or not, the inward GABA current was irreverent with KCC2 function. In contrast, when membrane potential was held at −40 mV, large amount of Cl^−^ intrudes when GABA_A_ receptors are activated and KCC2 is required to function to maintain the intracellular low level of Cl^−^ by continuously extruding Cl^−^. Indeed, in this condition, CTZ treated neurons showed significantly enhanced synaptic depression compared to the DMSO control (n = 8; P < 0.01; Fig. 3B). It indicated that the loss of membrane KCC2 due to convulsant treatment apparently impaired its function to extrude chloride and attenuated the inhibition of GABA_A_R.

Then, the change of the *E*_GABA_, which was determined by the measurement of the reversal potential of evoked IPSCs at the different holding potential in voltage clamp mode of perforation patch clamp, at the different time point after CTZ treatment was measured to study the temporal relation of the KCC2 function impairment. After continuous CTZ treatment for either 0.5 hr or 1 hr, there was a significant positive shift of the *E*_GABA_ after 1 hr CTZ treatment (CTZ 0 hr, −91.9 ± 2.9 mV *vs.* CTZ 1 h, −70.5 ± 7.4 mV, n = 5; P < 0.05; Fig. 3D), but no obvious change of the *E*_GABA_ after only 0.5 hr CTZ treatment (CTZ 0 hr, −83.1 ± 2.8 mV *vs.* CTZ 0.5 hr, −85.4 ± 4.9 mV, n = 5; P = 0.70; Fig. 3C), which is in consistent with our WB data. In addition, we also detected that CTZ incubation remarkably led to a depolarizing shift of *E*_GABA_ at 2 hr time point compared to DMSO control (DMSO, −70.8 ± 1.1 mV, n = 9 *vs.* CTZ, −60.9 ± 1.9 mV, n = 8; P < 0.001; Fig. 3E,F).

Since positive shift of *E*_GABA_ could be induced by either a decrease of KCC2 or an increase of NKCC1, we performed pharmacological experiments by using either furosemide (100 µM), a KCC2 preferable blocker, or bumetanide (10 µM), a selective NKCC1 inhibitor, to test their contribution to this *E*_GABA_ change. In accordance with the perforated patch recordings, small tip whole-cell recordings also revealed a significant depolarizing shifted of *E*_GABA_ after 2 hr CTZ treatment (DMSO, −62.3 ± 0.7 mV, n = 13 *vs.* CTZ, −56.2 ± 0.9 mV, n = 11; P < 0.001; Fig. 3G,H). According to that, following experiments were conducted under small tip whole-cell recordings. Consistent with the previous reported^27^, when furosemide was added into the ACSF to block KCC2 function, a significant depolarizing shift of the *E*_GABA_ was detected (FUR, −58.1 ± 1.6 mV, n = 7, P < 0.05; Fig. 3G and H), which mimicked CTZ effect. However, there was no effect on *E*_GABA_ was detected in inhibiting NKCC1 with bumetanide (BUM, −64.0 ± 1.2 mV, n = 9, P = 0.2; Fig. 3G,H). Thus, this result further indicated that the convulsant stimulation-induced depolarizing shift of *E*_GABA_ was likely largely dependent on the loss of KCC2 function but not due to the involvement of NKCC1.

In conclusion, our current results indicated that the functional reduction of KCC2 occurred before the formation of highly synchronized bursting discharges, suggesting that the lack of KCC2 might act as an important contributing factor in generating seizure related epileptiform bursting activities.

### Suppression of endogenous KCC2 expression induced spontaneous epileptiform bursting activities in cultured hippocampal neurons

**Figure 4:**
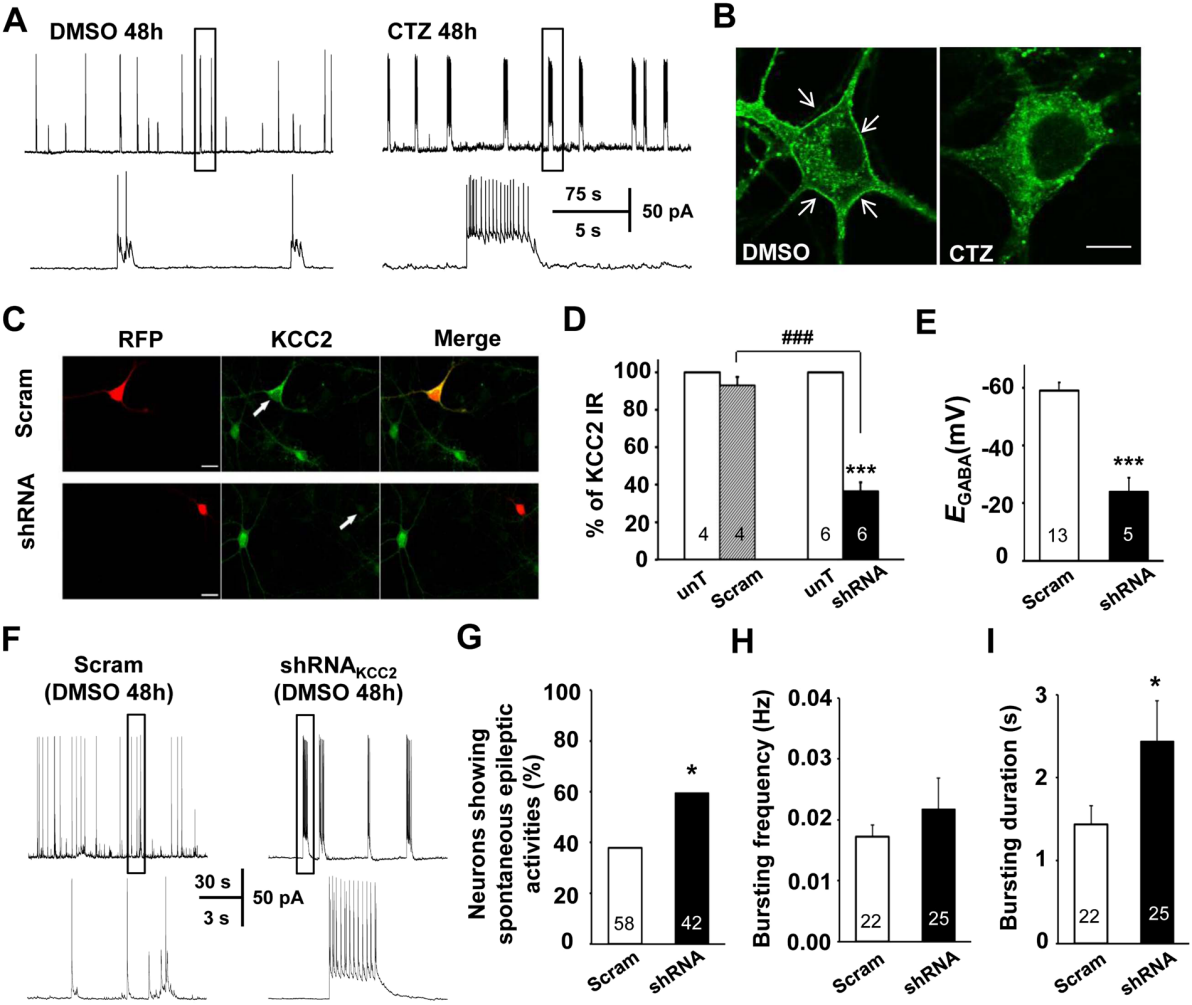
Knocking down of endogenous KCC2 expression induced spontaneous epileptiform activities in cultured hippocampal neurons. (**A**) Representative traces showing that recurrent epileptiform bursting firing could be induced after pretreatment with CTZ (5 μM, 48 hr) in a cultured pyramidal neuron. Epileptiform bursting firing in the form of a train of action potentials overlaying a large depolarization shift. Few epileptiform bursting firing were observed after treatment with vehicle DMSO. (**B**) Representative images showing membrane KCC2 immunostaining (arrows) in DMSO treated neuron, but the discontinuous clusters of KCC2 labeling in CTZ pretreated neuron membrane. Scale bars: 10 µm. (**C**) Representative images showing neurons transfected with plasmid containing either scrambled shRNA-RFP or shRNA_KCC2_-RFP, and subsequently immunolabelled with KCC2 antibody (in green). Scale bars: 20 µm. (**D**) KCC2 labeling was significantly decreased (***P < 0.001) in shRNA_KCC2_ transfected neurons, but not in scrambled shRNA transfected neurons (P = 0.17) in compared with those untransfected neurons from the same coverslip, indicating shRNA_KCC2_ is very effective to knock down endogenous KCC2 expression in neurons (^###^P < 0.001). (**E**) shRNA_KCC2_ transfection induced positive shifts in *E*_GABA_ (***P < 0.001). (**F**) Representative traces showing that shRNA_KCC2_ transfection facilitated the occurrence of spontaneous epileptiform bursting firing in cultured hippocampal neurons. (**G–I**) Bar plot showing downregulation of KCC2 expression by shRNA_KCC2_ significantly increased the percentage of neurons showing epileptiform bursting firing (*P < 0.05; **G**), and increased the bursting duration (*P < 0.05; **I**), but not the bursting frequency (**H**) in those bursting neurons.

To further investigate the role of KCC2 reduction on epileptiform bursting activity generation, we used cultured neuron model similar as previous reported^23, 24, 28^ for molecular manipulation of the KCC2 expression in neurons. Consistent with previous data^23^, after CTZ (5 µM) treatment for 48 hr, cultured hippocampal neurons showed abnormally synchronized bursting activities, with high-frequency action potentials overlaying large depolarizing shifts (>10 mV) (Fig. 4A). Moreover, immunocytochemistry staining showed endogenous membrane KCC2 expression was apparently decreased after CTZ treatment (Fig. 4B). In order to test whether epileptiform bursting activities were affected by the change of KCC2 expression level, we used a short-hairpin RNA (shRNA_KCC2_) to knock down endogenous KCC2 expression. Immunostaining with KCC2 antibody showed that shRNA_KCC2_ was very effective at knocking down endogenous KCC2 protein expression compared to scrambled shRNA (shRNA_KCC2_: 36.5 ± 4.6%, batches = 6, n = 31 *vs.* scrambled shRNA: 93.0 ± 4.4%, batches = 4, n = 19; P < 0.001), or untransfected neurons (100%, batches = 6, n = 52, P < 0.001; Fig. 4C,D). In this condition, we found that *E*_GABA_ of scrambled shRNA neurons was −59.0 mV ± 3.0 mV (n = 13), close to the reported Cl^−^ equilibrium potential of −52.3 mV in cultured neurons^29^. However, a significant positive shift of *E*_GABA_ was detected in shRNA_KCC2_ transfected neurons (−23.9 mV ± 4.9 mV, n = 5, P < 0.001; Fig. 4E). It suggested that suppression of KCC2 expression could effectively inhibited the capacity of Cl^−^ extrusion in neurons. As expected, even without convulsant stimulation, such as CTZ, the neurons with lowered KCC2 expression by shRNA_KCC2_ transfection with a significantly high percentage showed spontaneous epileptiform bursting activities in comparison with the neurons transfected with scrambled shRNA (scrambled shRNA: 38%, n = 58 *vs.* shRNA_KCC2_: 60%, n = 42, P < 0.05; χ2 test; Fig. 4F,G). In addition, among those bursting neurons, knocking down of KCC2 also significantly prolonged the duration of the bursting activities (scrambled shRNA: 1.4 s ± 0.2 s, n = 22 *vs.* shRNA_KCC2_: 2.4 s ± 0.5 s, n = 25, P < 0.05; Mann-Whitney test; Fig. 4H), but not much effect on the bursting frequency (scrambled shRNA: 0.017 Hz ± 0.002 Hz, n = 22 *vs.* shRNA_KCC2_: 0.022 Hz ± 0.005 Hz, n = 25, P = 0.83; Mann-Whitney test; Fig. 4I).

This result indicated that suppression of endogenous KCC2 expression and impairment of the KCC2 function alone are sufficient to induce neurons to generate spontaneous epileptiform bursting activities.

### Overexpression of KCC2 suppressed CTZ-induced epileptiform bursting activities in cultured hippocampal neurons

**Figure 5:**
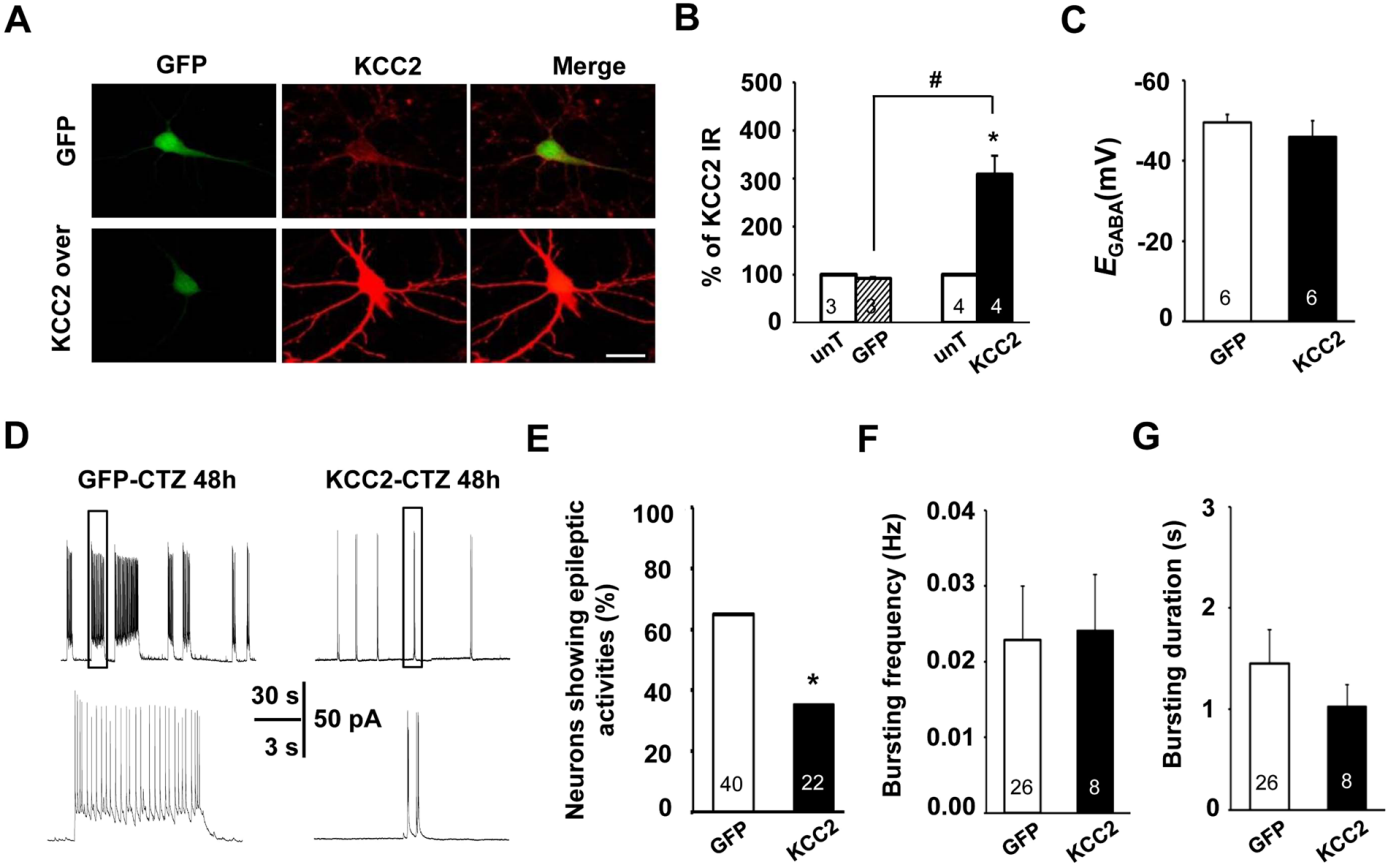
Overexpression KCC2 attenuated CTZ-induced epileptic activity in cultured hippocampal neurons. (**A**) Representative images showing neurons transfected with plasmid containing either GFP or KCC2-GFP, and subsequently immunolabelled with KCC2 antibody (in red). Scale bars: 20 µm. (**B**) Quantification of the density of KCC2 labeling. KCC2 density was significantly increased in KCC2-GFP transfected neurons compared to either the untransfected neurons from the same coverslip (*P < 0.05) or the GFP control neurons (^#^P < 0.05). (**C**) Group data showing there was no significant change of *E*_GABA_ after KCC2 overexpression (P = 0.4). (**D**) Sample traces showing overexpression of KCC2 in neurons suppressed CTZ-induced epileptiform bursting firing. (**E–G**) Bar histograms showing KCC2 overexpression significantly decreased the percentage of neurons having epileptiform bursting firing compared to the GFP neurons after 48 h CTZ pretreatment (*P < 0.05; **E**), but not affected either the bursting frequency (**F**) or the bursting duration (**G**) in those bursting neurons.

To test whether sufficiency of KCC2 could exert suppressive effect on CTZ-induced epileptic activity, we used KCC2-pIRES2-EGFP plasmid to overexpress KCC2 before CTZ treatment in cultured hippocampal neurons. Immunostaining with KCC2 antibody showed that KCC2 expression, compared to the untransfected neurons, was successfully upregulated for over 3-folds in KCC2-pIRES2-EGFP transfected neurons (KCC2-GFP: 309.2 ± 38%, batches = 4, n = 20, P < 0.05; Fig. 5A,B) but not in EGFP transfected neurons (GFP: 92.3 ± 3.5%, batches = 3, n = 13, P = 0.17; Fig. 5A,B). In these neurons, *E*_GABA_ results showed that there was no remarkable difference between KCC2 overexpression neurons and GFP alone neurons (GFP: −49.5 mV ± 2.0 mV, n = 6 *vs.* KCC2-GFP: −45.9 mV ± 4.0 mV, n = 6, P = 0.4; Fig. 5C). Therefore, KCC2 overexpression did not affect chloride extrusion capacity. However, in those KCC2 overexpression neurons, the proportion of the neurons showing CTZ induced epileptiform bursting activities was significantly decreased (GFP: 65%, n = 40 *vs.* KCC2-GFP: 35%, n = 22, P < 0.05; χ^2^ test; Fig. 5D,E). Overexpression of KCC2 had no effect on either bursting frequency (GFP: 0.023 Hz ± 0.007 Hz, n = 26 *vs.* KCC2-GFP: 0.024 Hz ± 0.007 Hz, n = 8, P = 0.34; Mann-Whitney test; Fig. 5F) or duration in those bursting neurons (GFP: 1.5 s ± 0.5 s, n = 26 *vs.* KCC2-GFP: 1.0 s ± 0.2 s, n = 8, P = 0.30; Mann-Whitney test; Fig. 5G).

This result demonstrated that KCC2 overexpression in cultured hippocampal neurons significantly suppressed the probability of neurons to be induced to have epileptiform bursting activities by the convulsant CTZ. It suggested that the KCC2 level is important in the seizure induction, maybe essential for the epileptiform bursting generating in the neurons.

### Knocking down of KCC2 expression level facilitated epileptic seizure *in vivo*

**Figure 6:**
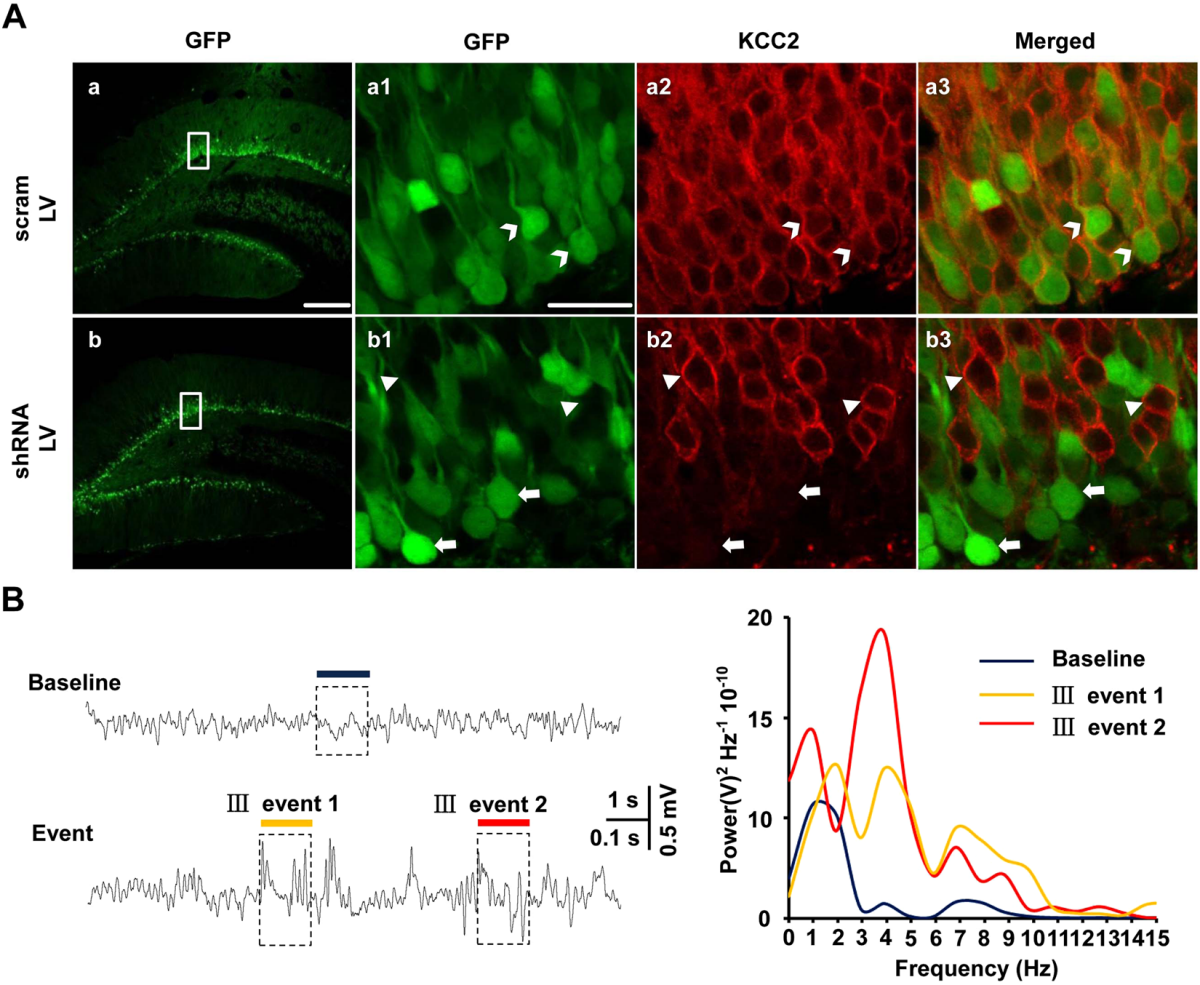
Knocking down of KCC2 expression in DG induced seizure behavior in freely moving adult rat. (**A**) Sample confocal scanning images of virus expression and KCC2 immunolabeling in dentate gyrus (*DG*). (a,b) Lenti-virus packed with either scram vector with EGFP as control (a) or shKCC2-EGFP (b) expressed in the rats DG area 14 days after injection. Scale bars: 200 µm. (**a1-b1**) Enlarged view of framed area in a-b. Scale bars: 20 µm. (**a2-b2**) Images of KCC2 labeling (red). (**a3-b3**) Merged images showing co-labeling of EGFP with KCC2 was observed in EGFP infection rat (a3, white open arrowhead), but hardly any in shKCC2 infected rat DG neurons (b3, white arrow). Non infection neurons were labeled with KCC2 signal in shKCC2 infected rat (b3, white closed arrowhead). (**B**) Representative EEG recordings from shKCC2-EGFP virus injection rats, juxtaposing baseline and abnormal events corresponding to spontaneous Racine III seizure behavior (Left upper and bottom). A power spectrum from framed representative baseline and abnormal events (Right) showing the peak frequency difference between shKCC2-EGFP infection induced epileptic episode and the baseline of the EEG.

Since knocking down of KCC2 expression in cultured hippocampal neurons could facilitate the seizure related epileptiform bursting activities, we further investigated whether downregulation of KCC2 *in vivo* could be prone to generate spontaneous seizure activity. We used lenti-virus containing with either shKCC2-EGFP or the scrambled shRNA vector with EGFP as control to knock down KCC2 expression in hippocampal dentate gyrus area (DG). Confocal images showed that both shKCC2-EGFP and scram-EGFP virus successfully infected DG cells. Brain sections immunostaining with KCC2 antibody showed GFP positive cells were co-labeled with KCC2 signal in scrambled shRNA control rat, while none or little co-labeling of GFP with KCC2 signal was observed in shKCC2 rat (Fig. 6A). Therefore, data indicated that the membrane KCC2 protein expression was efficiently knocked down by shKCC2-EGFP in DG cells *in vivo*. In three rats, more than two injection positions, bilaterally, were observed to be successfully infected with shKCC2-EGFP virus. Short lasting abnormal EEG events with corresponding spontaneous Racine III seizure behaviors were caught by EEG/video monitoring. The power analysis of the representative EEG traces revealed that the frequency peak of the ictal like EEG activities was at around 3–5 Hz (Fig. 6B), which was in agree with above result that the mean frequency for Racine III was at 3.6 ± 0.3 Hz in CTZ-induced seizure animals (see Fig. 1F). No abnormal activity was detected in two virus infection control rats, although the recording time was similar. Above results indicated that knocking down endogenous KCC2 alone could induce to generate epileptiform bursting EEG and spontaneous seizure in freely moving rats.

## Discussion

Our main findings are: 1) ictal-like epileptiform bursting activities in EEG could be directly correlated to the seizure behavior in freely moving rats in CTZ seizure model, and significant KCC2 downregulation only occurred in Racine score III and above seizure behavior rats with ictal-like epileptiform bursting activities; 2) the downregulation of the membrane KCC2 expression and Cl^−^ extrusion function occurred much earlier than ictal-like epileptiform bursting activities in hippocampal slices; 3) knocking down of KCC2 expression induced the occurrence of spontaneous epileptiform bursting activities *in vitro*, and seizure behavior with ictal-like epileptiform bursting EEG activities *in vivo*; and 4) upregulation of KCC2 expression inhibited convulsant induced epileptiform bursting activities *in vitro*. These findings showed that KCC2 downregulation during epileptogenesis played a facilitation role in generating ictal-like epileptiform bursting activities in neurons, hence to evoke seizure.

Researches have found that KCC2 was downregulated in various seizure animal models. Hippocampal KCC2 immunostaining was reduced 6 hr after kindling-induced seizures^30^ and plasma membrane KCC2 in dentate gyrus was significantly downregulated for 1 and 2 weeks after pilocarpine-induced status epilepticus^13^. Consistent with those previous findings *in vivo*, reduction of hippocampal membrane KCC2 have also been observed in our current study in CTZ-induced seizure model. MRS IV or above behaviors are used to estimate whether acute seizure model is well established. In this study, MRS IV or above seizure behaviors were noticed to correspond with the synchronized HFHA bursting events of EEG by monitoring behavior and EEG recording simultaneously. Interestingly, we found that characteristic epileptiform discharges appeared in MRS III animals as well. Moreover, KCC2 reduction was also detected only in MRS III or above, but not in MRS II or below animals. These results suggested that epileptiform bursting activities were closely linked to seizure behaviors, and could be regarded as an indicator to investigate the temporal relation between seizure behavior and protein (such as KCC2) expression change. In this study, development of neuronal network activities from interictal-like activity to ictal-like bursting discharges could be induced in both Mg^2+^ free ACSF and CTZ model in hippocampal slices preparation. Similar to previous studies, interictal-like activity induced by Mg^2+^ depletion led to reduced KCC2 expression^4, 31^. Previous research has reported that spontaneous interictal-like, but not ictal-like activities, induced downregulation of KCC2 in a subpopulation of subicular principal neurons from adult patients with temporal lobe epilepsy^32^. In agree with the human result, we found that downregulation of membrane KCC2 had occurred (<1 hr) before ictal-like bursting discharge occurrence, but along with the interictal-like spike discharges in the hippocampal neurons. Furthermore, we also found the occurrence of depolarizing shift of *E*_GABA_ was earlier than the epileptiform bursting discharge generation. Since the change in *E*_GABA_ is attributed to the altered KCC2-mediated Cl^−^ extrusion capacity, It suggested functional KCC2 reduction was in advance of epileptiform bursting discharges. In addition, NKCC1, another chloride cotransporter mainly expressed in the neuron in neonatal stage, have been reported to be upregulated during seizure. However, our WB blot results from the same sample as detected the KCC2 downregulation showed no change of the NKCC1 expression in the hippocampal neuronal membrane. Taken together, our findings by first time sorted out the temporal relationship between the KCC2 downregulation and the generating of ictal-like epileptiform bursting activities, which indicating that the impairment of KCC2 was not just the consequence of the epileptic seizure, but might be a key factor to facilitate the epileptiform ictal-like bursting activity happening, and hence the seizure behavior.

For the purpose to figure out the functional role of KCC2, increasing researches take advantage of molecular approaches to manipulate endogenous KCC2 expression both *in vitro* and *in vivo*. *In vitro*, silencing of endogenous KCC2 in cultured neurons reduces neurotoxic resistance^33^. *In vivo*, KCC2 homozygous mutant mice could not survive for long time after birth^34^. Frequent seizure episodes presented in those mice might result in the postnatal morality^35^. Dysfunction of KCC2 by biallelic mutations might induce migrating focal seizures^36^. Besides, although no abnormal phenotype was showed in heterozygote mice, a higher susceptibility for seizure activity was observed in comparison with wild-type ones^35^. In this study, knocking down of the endogenous KCC2 using shRNA_KCC2_ increased the risk to generate spontaneous bursting activities. Instead of that, overexpression of KCC2 even suppressed the ratio of neurons showing bursting activities induced by convulsant. But, it is not known whether reduction of functional KCC2 would lead to the occurrence of the epileptiform activities or seizure behaviors in animal. To work out the question without affecting neuronal maturation, shKCC2 lentivirus was adopted to knock down KCC2 in dentate gyrus in adult rats. As expected, spontaneous seizure episodes and associated HFHA EEG were detected in shKCC2 group rats with well infected neurons bilaterally in DG area, but not in virus infection control group rats. This result strongly supported our view that hippocampal KCC2 expression downregulation could induce spontaneous seizure occurrence.

Thus, the remaining question was how the KCC2 was downregulated at the time point (<1 hr) earlier than the epileptiform bursting activities occurrence after CTZ treatment in hippocampal slices. The membrane stability and the function of KCC2 is subjected to be regulated by residues phosphorylation, such as serine 940 (S940) and tyrosine (Tyr-1087)^37–39^. Since phosphorylation of S940 maintains the stability of KCC2 on the cell surface and the co-transporter activity, most of the studies were focused on S940 residues of KCC2^20, 32, 37^. Dephosphorylation of S940 was reported to involve in glutamate induced activity dependent downregulation of membrane KCC2 and *E*_*GABA*_ positive shifts^20^. Recent research even demonstrated that S940 mutant accelerated the latency and lethality of status seizure induced by KA *in vivo* ^40^. Here, we discovered that there was a significant dephosphorylation of S940 (reduced ratio of membrane pS940 KCC2/total membrane KCC2) occurred as early as at 1 hr after CTZ treatment, along with the downregulation of the membrane KCC2 level, earlier than the first occurrence time point for ictal-like bursting activities of the neurons. Considering the importance of pS940 on surface KCC2 stability and the significant reduction of the pS940 at 1 hr CTZ treatment before epileptiform bursting activities, we hypothesized that the dephosphorylation of the pS940 induced the instability of the membrane KCC2 and caused the downregulation of the membrane KCC2 in the early stage of seizure induction. Thus, maintaining of the stability of the membrane KCC2, such as preserving the pS940 residues of KCC2, likely would play a crucial role to limit the epileptogenesis under pathological condition^40^.

Taking together, this study suggested that the reduction of membrane KCC2 during increased neuronal activity after pathologic, such as convulsant stimulation, condition, further interrupted the imbalance of excitation and inhibition by suppressing the GABA inhibitory function, drove the neuronal network to develop bursting activity, and finally led to the formation of ictal-like epileptiform bursting neuronal activities and seizure. It provides a new avenue for interrupting seizure occurrence by either blocking of KCC2 downregulation or increasing the upregulation during the early phase of pathologic conditions. Thus, protection of KCC2 downregulation could serve as a new target for developing novel anti-epileptic drugs.

## Methods

### Ethics statement

All the animal experiments were approved by the Local Committees of the Use of the Laboratory Animals, Fudan University (Shanghai, China) and were carried out in accordance with the guidelines and regulations of National Natural Science Foundation of China animal research.

### Cell culture and transfection

Embryos were prepared from day 18 embryonic Sprague Dawley (SD) rats under ether anesthesia similar to our reported work^41–43^. Briefly, hippocampus was dissected out and digested in 0.05% trypsin-EDTA solution for 15–20 min in 37 °C incubator. After digestion, single cells were subsequently isolated, collected by centrifuging at 1000 rpm for 8 minutes and resuspended in neuronal medium. Then cells were plated on poly-D-lysine coated coverslips at the density of 40 k cells/cm^2^ in neurobasal medium supplemented with 2% B27 (GIBCO, Life Technologies). Cell cultures were used for experiments between 14 and 21 d *in vitro* (DIV). Transfections were carried out at DIV 9–10, using a modified Ca^2+^-phosphate transfection protocol similar as previously reported^28^.

### Plasmid constructs

KCC2 cloned into pIRES2-EGFP and small hairpin RNA plasmid interfering with the expression of KCC2 (shRNA_KCC2_), were designed by Shanghai Genechem Co. Ltd. The target sequences (GCCATTTCCATGAGCGCAA) were inserted into pGCsi-U6-RFP vector to generate the shRNA_KCC2_ construct and a non-silencing scrambled sequence as a control shRNA vector.

### Brain slice preparation

Male SD rats, aged p35–40, were anesthetized with sodium pentobarbital (60 mg/kg, i.p.). After decapitation, brains were dissected quickly and placed in ice-cold artificial cerebrospinal fluid (ACSF) containing (in mM) 119 NaCl, 2.5 KCl, 2.5 CaCl_2_, 1.3 MgSO4, 1 NaH2PO4, 26.2 NaHCO3, and 11 Glucose, pH 7.3 (~300 mOsM). Slices (300–350 μm) were collected with a vibratome (Leica Instruments, German) and maintained in continuously oxygenated ACSF for at least 30 min at 33 °C and then at room temperature for 1 hour before recording. Individual slices were transferred to a submerged recording chamber visualized with infrared optics microscope (Nikon, Japan).

### Electrophysiology recordings

Spontaneous field potentials were recorded in dentate gyrus using microelectrodes filled with normal ACSF. In evoked response experiments, whole-cell patch recordings were made from CA1 pyramidal neurons about 100 μm away from the stimulation electrode in the schaffer collateral (SC). 3–6 MΩ patch pipettes were filled with following internal solution (in mM): 125 K-Gluconate, 15 KCl, 10 HEPES, 0.5 EGTA, 2 Mg-ATP, 0.5 Na-GTP and 10 Na_2_-Phosphocreatine with pH 7.3 (~300 mOsM). In the presence of DNQX (15 μM) and DL-AP5 (50 μM), repetitive GABAergic synaptic activities were conducted in conditions that either Cl^−^ efflux (holding potential at −80 mV) or influx (at −40 mV)^27^. The SC pathway was stimulated with a high frequency paradigm (20 Hz, 24 pulses). For gramicidin perforated patch clamp recording, electrodes tips were filled with internal solution (140 KCl, 2 MgCl_2_, 10 HEPES, 2 EGTA, 2 Mg-ATP, 0.5 Na-GTP, 10 Na_2_-Phosphocreatine, pH 7.3) first and then back-filled with internal solution containing gramicidin (40 μg/ml). Membrane channels permeable to monovalent cations but not to Cl^−^ are formed by gramicidin^44^, so intracellular Cl^−^ concentrations will not be perturbed. At stepped holding membrane potentials (10 mV increments from −80 to −40 mV), single stimulus evoked GABA current was recorded. Linear regression was used to calculate a best-fit line for the voltage-current relationship. The voltage value cross with the x-axis was taken as the *E*_GABA_. Besides perforated patch recordings, whole-cell recordings using small pipette tip (10–14 MΩ) was also used to evaluate the *E*_GABA_, as previously reported^27, 45^. In cultured neuron experiments, after 48 hr pre-treatment with CTZ (5 μM) or DMSO (0.1%), coverslip was transferred to recording bath solution contained (in mM): 128 NaCl, 30 Glucose, 25 HEPES, 5 KCl, 2 CaCl_2_, and 1 MgCl_2_, pH 7.3, ~320 mOsM.). *E*_GABA_ was evoked by puffing GABA (200 μM) on to the soma at stepped holding membrane potentials (10 mV increments from −80 to −40 mV) by using gramicidin-perforated patch clamp recordings. CTZ-induced epileptiform activities were performed in whole cell current clamp mode as described previously^23, 24, 28^. Patch pipettes were filled with following internal solution (in mM): 125 K-Gluconate, 10 KCl, 2 EGTA, 10 Hepes, 10 Tris-Phosphocreatine, 4 MgATP, 0.5 Na_2_GTP, pH 7.3, ~305 mOsM. The membrane potential was maintained at −70 mV by injecting current during recording. The definition of epileptiform bursting activity is that at least five consecutive action potentials is overlaying on top of the large depolarization shift (≥10 mV, ≥300 ms)^23^. Electrical signals were digitized with Digidata 1440A and Multiclamp 700B amplifier (Molecular Devices) using pCLAMP 10.2 software.

### Stereotaxic lentivirus injection

Anesthetized rats (p35-40, male) were placed in a stereotaxic apparatus with homeothermic blanket. Total four positions of bilateral dentate gyrus were injected as follows: (AP −3.6 mm, ML ± 2.0 mm, DP −3.6 mm); (AP −4.4 mm, ML ± 2.4 mm, DP −3.6 mm). Either shKCC2-RNAi-LV(Ubi) (2 × 10^9^ TU/ml, Genechem) or scram control FU-RNAi-NC-LV (5 × 10^9^ TU/ml) was infused (2 μl at 0.2 μl/min) using Hamilton syringe (26 gauge needle). 10 min later after infusion, the syringe was slowly withdrawn. After surgery, animals were allowed to recover for 7 days before the EEG and video recording.

### EEG recording and behavior assays

Behavioral seizures in freely moving rats combination with electroencephalograph (EEG) after CTZ induction was recorded as described previously^21^. In generally, anesthetized adult rats were placed in stereotaxic apparatus with homeothermic blanket. A guide cannula (22GA) was embedded left lateral ventricle (AP −0.3 mm, ML 1.3 mm, DP −4.0 mm) for CTZ microinjection. Two stainless steel screws were fixed at the skull above the hippocampus (AP −3.8 mm and ML 2.0 mm) as recording electrode and above the contralateral forehead (AP 1.5 mm and ML 1.5 mm) as reference electrode respectively. A micro-connector was connected to electrodes and immobilized to skull with dental cement. Animals were allowed to recover for at least 5 days after the surgery and then habituate for at least 20 min before DMSO (vehicle control) and CTZ injection (0.125 μmol in 5 μl, i.c.v.). Seizure behaviors as well as EEG performance were simultaneously recorded within 1 hr after injection and terminated by sodium pentobarbital (i.p.). The EEG signals were amplified (1000 times) and filtered (0.3–1 kHz) with NeuroLog System (Digitimer Ltd., Hearts, UK), digitized with CED Micro 1401 (Cambridge Electronic Design, Cambridge, UK) and recorded with Spike 2 software. The modified Racine’s score (MRS)^46^ was used to scale the CTZ-induced seizure behaviors: MRS I-II: facial clonus and head nodding; MRS III: unilateral forelimb clonus; MRS IV: rearing with bilateral forelimb clonus; MRS V: rearing and falling; MRS VI: running or bouncing seizures; MRS VII: tonic hind limb extension.

### Immunostaining

For Immunocytochemistry, cells were fixed by 4% paraformaldehyde (PFA, Sigma) for 10–12 min. After rinses in Tris-buffered saline (TBS), cells were permeabilized and blocked for 2 hr in 0.2% Triton X-100 (Sigma) and 10% normal donkey serum (NDS, Millipore) in TBS at room temperature (RT), then incubated in primary antibody (rabbit anti-KCC2, against residues 932–1043 of the rat KCC2, #07-432, 1:300, Millipore) diluted in 10% NDS at 4 °C overnight. After rinses, cells were incubated with corresponding secondary antibodies (donkey anti-rabbit conjugated to Alexa Fluor 594 or 488, 1:300; Molecular Probe) diluted in 10% NDS at RT. Finally, cells were rinsed and coverslipped with ProLong Gold antifade reagent (Molecular Probe). Images were acquired with confocal scanning microscope with 60x oil immersion objective (Olympus). For immunohistochemistry, deeply anesthetized rats were perfused transcardially with saline, followed by 4% PFA. Brains were removed and stored in PFA overnight in the refrigerator. After dehydration in 30% sucrose for cryoprotection, 30 μm-thick coronal hippocampal sections were cut. The sections including the injection position were chosen and thoroughly rinsed in TBS, permeabilized and blocked for 2 hr in 0.2% Triton and 10% NDS in TBS at RT. Then the sections were incubated with primary antibody (anti-KCC2, 1:300) diluted in 10% NDS at 4 °C overnight. After rinses, sections were incubated with the secondary antibodies for 2 hr (Alexa Fluor 594, 1:300) diluted in 10% NDS at RT. Sections were rinsed at least 30 min, mounted on slides and coverslipped with antifade reagent. Sections were acquired with AZ-C2 with a 5x objective and A1R with a 25 × objective (Nikon).

### Western blots

Hippocampus were dissociated from brain slices under a dissecting microscope (Leica), then homogenized in pre-cooled lysis buffer (#K268-50, Biovision) quickly. The plasma membrane protein fractions were extracted by using Membrane Protein Extraction Kit (#K268-50 Biovision), dissolved in 0.5% Triton X-100 in PBS, and then bathed in 45 °C with SDS sample buffer for 45 min. Membrane proteins were separated by SDS-PAGE, electrophoretically transferred to Poly vinylidene fluoride membranes (Millipore), then incubated with primary antibodies rabbit anti-KCC2 (#07-432, 1:1000, Millipore), anti-NKCC1 (#14581, 1:1000, Cell Signaling Technology, CST), β-actin (#4967, 1:1000, CST) in 5% skimmed milk - TBS-T solution overnight at 4 °C, followed by incubation with goat anti-rabbit (Jakson) as secondary antibody in TBS-T buffer. Phosphorylated residue S940 was determined by using rabbit pS940 antibody (#32788,1:500, Rockland) as primary antibody and goat anti-rabbit (Jakson) as secondary antibody. After stripping out the pS940 antibody, the membrane protein was re-incubated with mouse anti-KCC2 (SAB5200222, 1:1000, Sigma) and then goat anti-mouse (Jakson) as secondary antibody. Bands were visualized by using an ECL detection system (Pierce). The immunoreactivity of individual band was measured by Image Pro Plus and normalized to β-actin.

### Statistical analysis

Statistical analysis was performed by SPSS 19.0 using one sample t-test, the unpaired student’s t-tests to compare two experimental groups. Corresponding nonparametric analysis (i.e. Independent-Samples Mann-Whitney) would be used if data were not normally distributed or not homogeneity of variance. Qualitative differences between two groups were analyzed by chi-square (χ2) test and Fisher’s exact. All data was presented as mean ± SEM. Significance level was defined as P value less than 0.05 and abbreviated as follows: *P < 0.05, **P < 0.01, ***P < 0.001.

## Electronic supplementary material

Supplementary information

Supplementary video (S-1a) left rat showing MRS I-II seizure behavior

Supplementary video (S-1b) showing MRS III seizure behavior

Supplementary video (S-1c) left rat showing MRS IV-VII seizure behavior
