# Supplementary material for: KCC2 downregulation facilitates epileptic seizures
Source: Sci Rep. 2017 Mar 13;7:156. doi: 10.1038/s41598-017-00196-7 (PMC5427808; doi:10.1038/s41598-017-00196-7)
Supplement: Supplementary file 1 — Supplementary information [file 41598_2017_196_MOESM1_ESM.pdf]

Supplementary information

KCC2 downregulation facilitates epileptic seizures

Lulan Chen[1, #], Li Wan[1, #], Zheng Wu[1], Wanting Ren[1], Yian Huang[1], Binbin Qian[1], Yun Wang[1, *]

[1]Institutes of Brain Science, State Key Laboratory for Medical Neurobiology, Collaborative Innovation Center for Brain Science, Fudan University, Shanghai 200032, China

[#]equal contribution

* Corresponding authors:
Dr Yun Wang: Institutes of Brain Science and State Key Laboratory for Medical Neurobiology, Fudan University, Shanghai 200032, China
Tel: (+86) 021 54237871; Fax: (+86) 021 54237643;
Email: yunwang@fudan.edu.cn

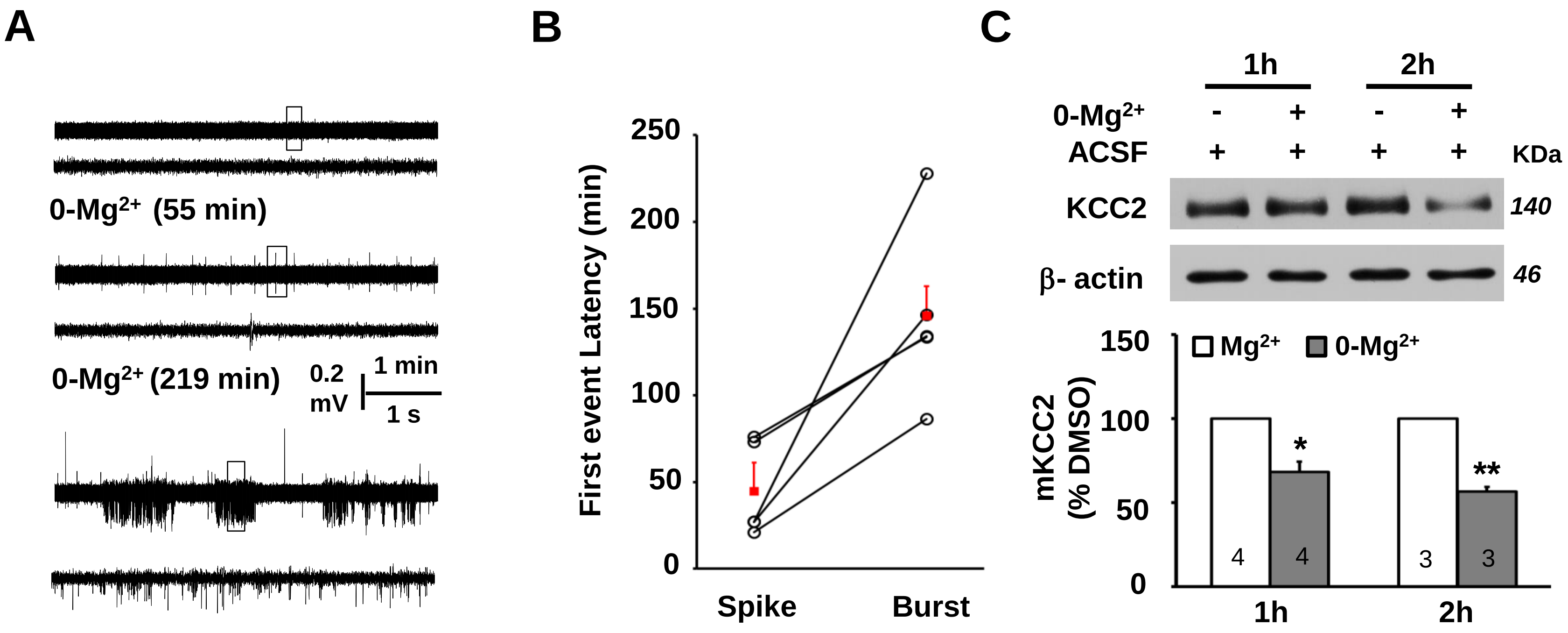

Supplementary Figure 1. Reduction of membrane KCC2 expression involved in the progress of epileptiform activity after 0-Mg^{2+} application in hippocampal slices. (**A**) Extracellular field potential recording of epileptiform activities in DG granule cell layer induced by 0-Mg^{2+} ACSF. Sample traces showing baseline (upper), interictal-like spike discharge (middle) or bursting activity (bottom) at different time points in 0-Mg^{2+} solution. (**B**) Dot plot showing the individual and group data (in red) of the latency of interictal-like spike and ictal-like bursting activities after 0-Mg^{2+} application. (**C**) Sample western blot showing membrane KCC2 expression was significantly reduced at 1 and 2 hr after 0-Mg^{2+} treatment. The gels were run under the same experimental conditions and were cropped around 140 and 46 KDa. The full-length gels were presented in Supplementary Figure 4. Bar histogram showing the quantification of KCC2 expression normalized to control (*$P < 0.05$, **$P < 0.01$).

Full-length gels

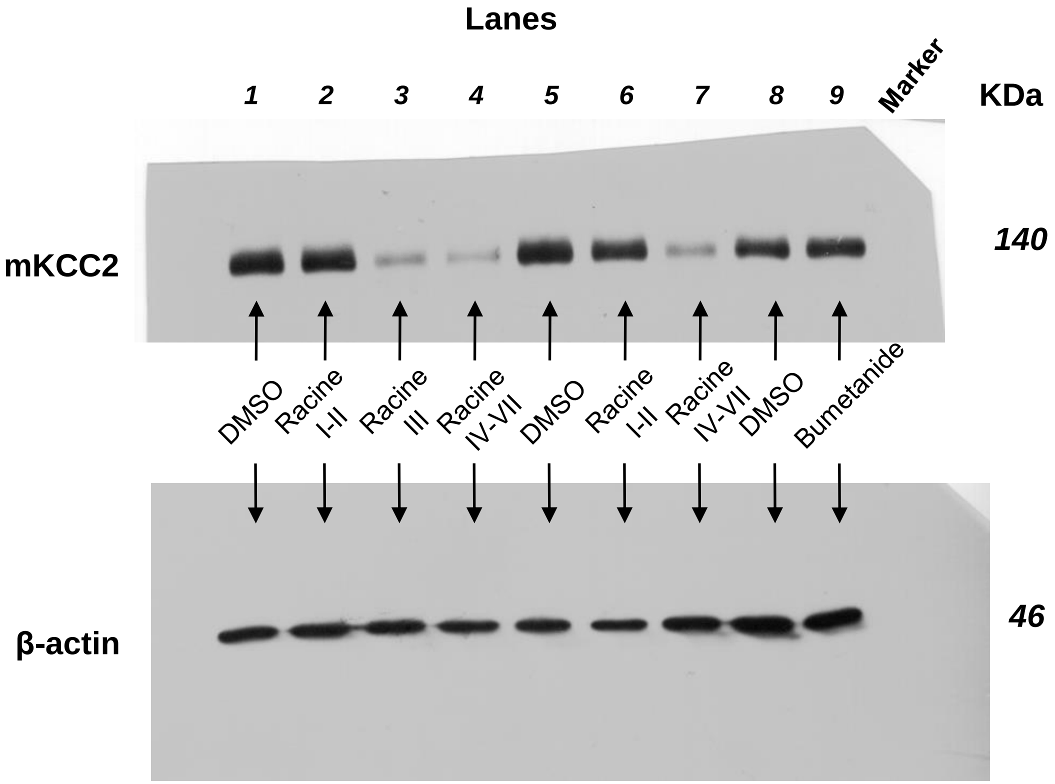

Supplementary Figure 2. Membrane KCC2 expression in different seizure level animals. Lanes (1-4) were cropped individually around 140 KD for KCC2 and 46 KD for β-actin, and represented in Fig. 1G. Samples were loaded onto the gel and run under same experimental condition. Photograph was gained by film sensation and scanned by a high resolution scanner.

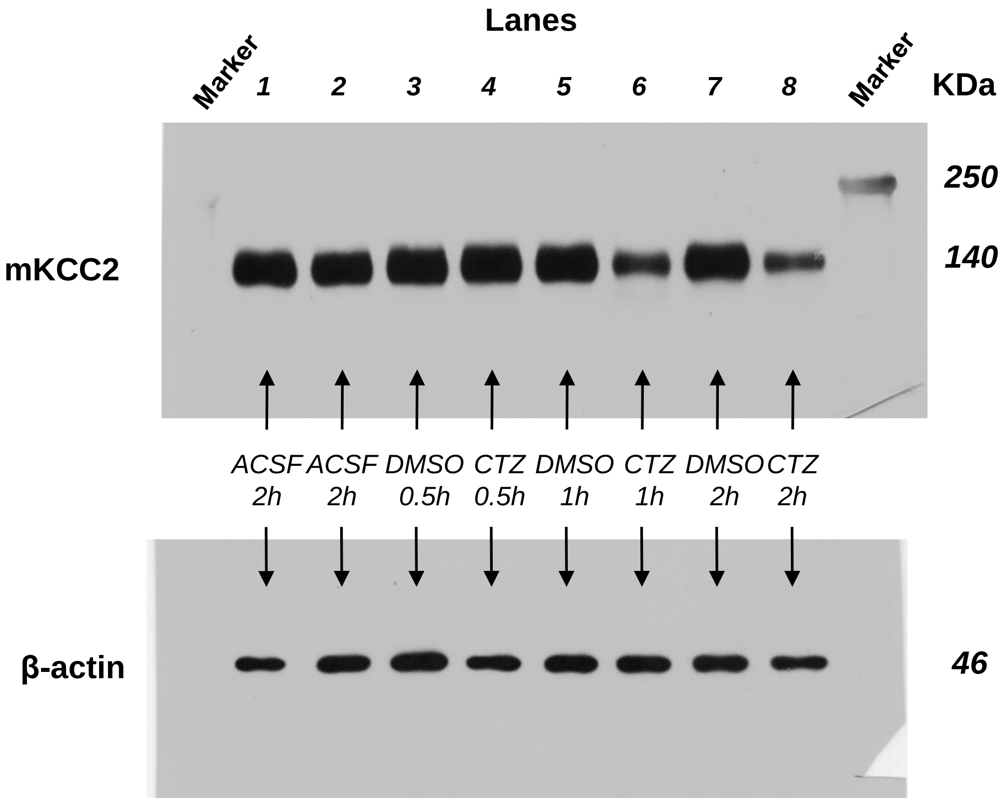

Supplementary Figure 3. Membrane KCC2 expression in hippocampal slices after DMSO or CTZ treatment. Lanes (3-8) were cropped individually around 140 KD for membrane KCC2 and 46 KD for β-actin, and represented in Fig. 2E. Samples were loaded onto the gel and run under same experimental condition. Photograph was gained by film sensation and scanned by a high resolution scanner.

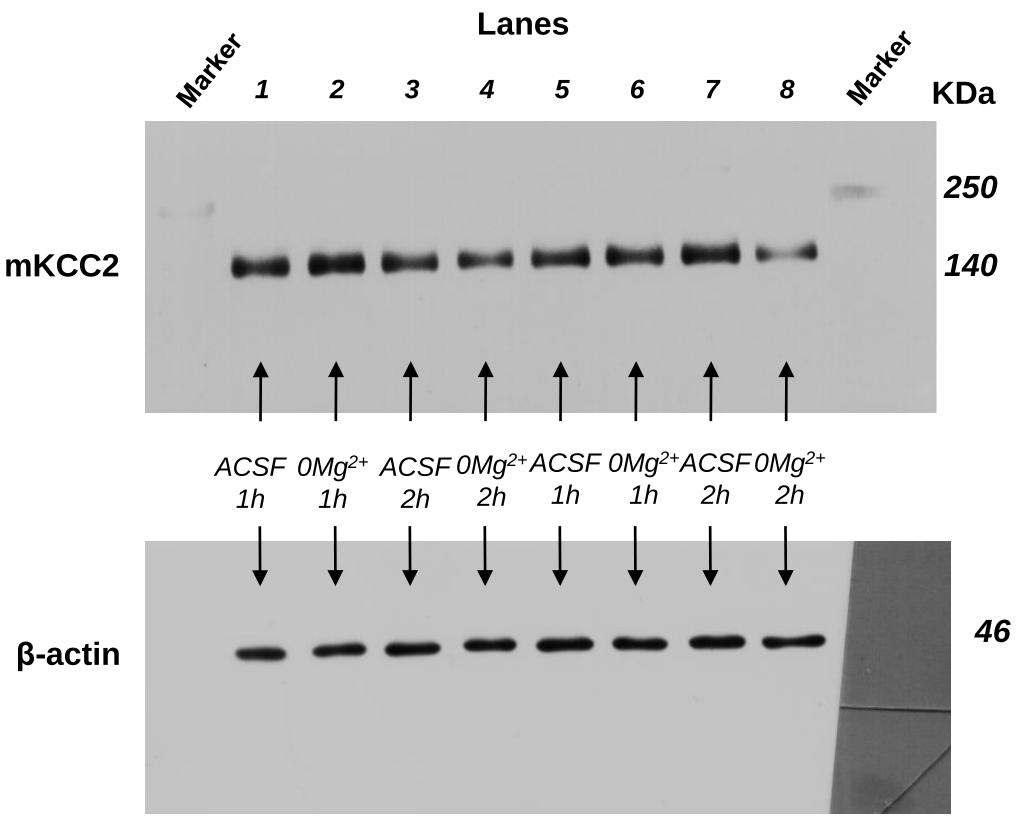

Supplementary Figure 4. Membrane KCC2 expression in hippocampal slices after 0-Mg^{2+} application. Lanes (5-8) were respectively cropped around 140 KD for KCC2 and 46 KD for β-actin, and represented in supplementary Fig. 1C. Samples were loaded onto the gel and run under same experimental condition. Photograph was gained by film sensation and scanned by a high resolution scanner.

.

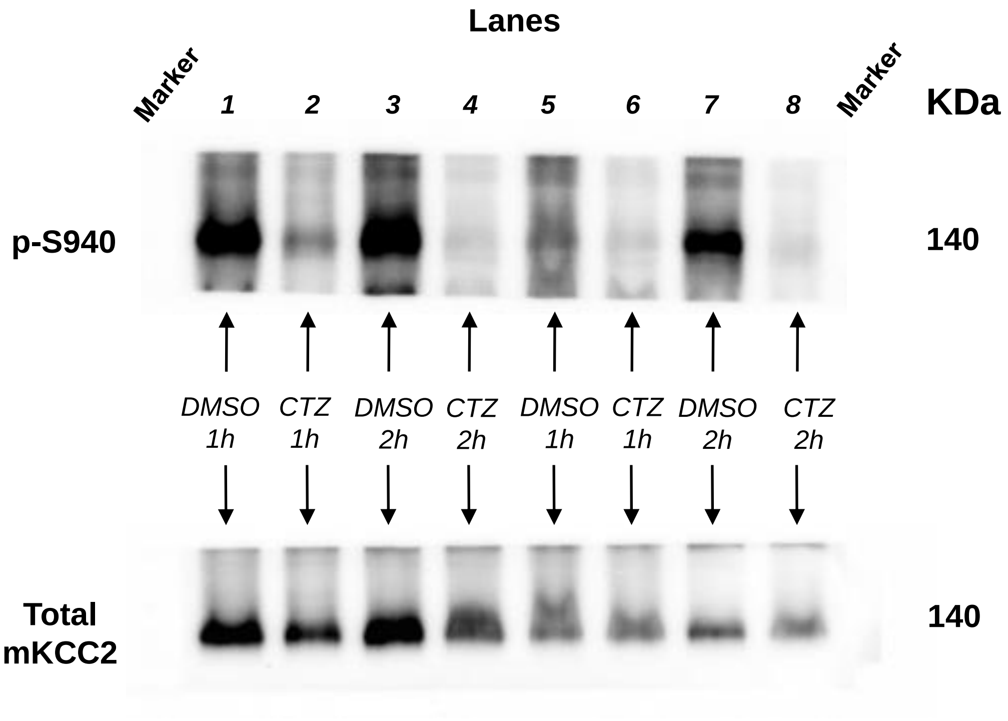

Supplementary Figure 5. Membrane p-S940 KCC2 and total membrane KCC2 expression in hippocampal slices after DMSO or CTZ treatment. Lanes (1-4) were cropped respectively around 140 KD for pS940 and KCC2 after stripping out p-S940, and represented in Fig. 2E. Samples were loaded onto the gel and run under same experimental condition. This graphics was obtained by a Fluochem E system, which is for superior analysis of chemiluminescent western blots.

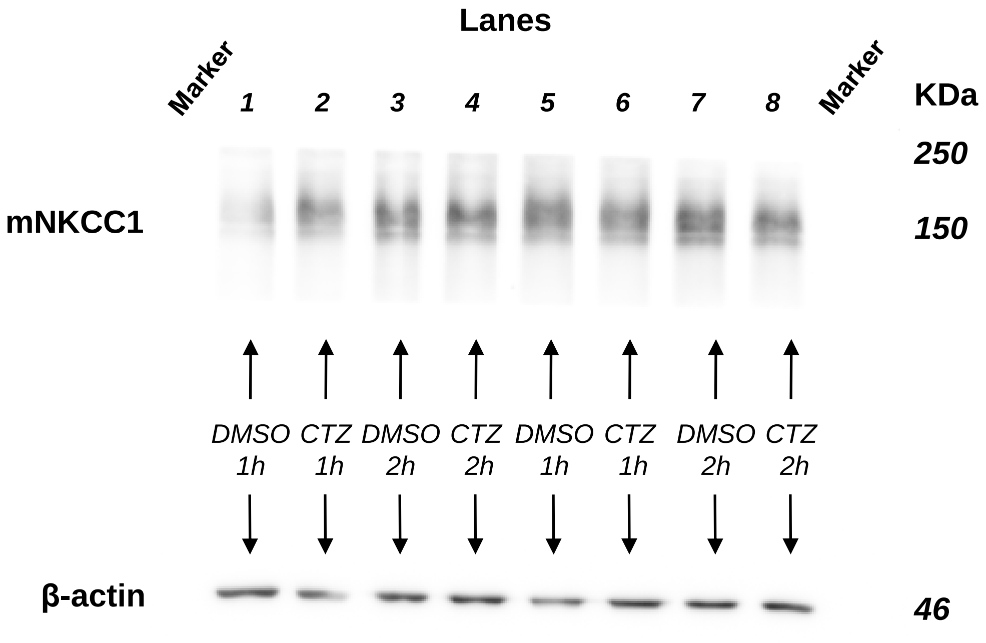

Supplementary Figure 6. Membrane NKCC1 expression in hippocampal slices after DMSO or CTZ treatment. Lanes (5-8) were respectively cropped around 150 KD for NKCC1 and 46 KD for β-actin, and represented in Fig. 2F. Samples were loaded onto the gel and run under same experimental condition. This graphics was obtained by a Fluochem E system, which is for superior analysis of chemiluminescent western blots.

Supplementary Video 1. Seizure behaviors with various maximum seizure levels were recorded after sub-maximal dose of CTZ injection. (a) Representative video (left rat) showing MRS I-II seizure behavior recording along with the window area of representative EEG recording in Fig.1C. **(b)** Representative video showing MRS III seizure behavior recording accompanied with the window area of representative EEG recording in Fig.1D. **(c)** Representative video (left rat) showing MRS IV-VII seizure behavior recording accompanied with the window area of representative EEG recording in Fig.1E.
